# Attitudes and Practices Surrounding Opioid Prescriptions following Open Reduction Internal Fixation of Distal Radius and Ankle Fractures: A Survey of the Canadian Orthopaedic Association Membership

**DOI:** 10.1155/2023/9968219

**Published:** 2023-09-07

**Authors:** Jihad A. Abouali, Evan D. Curd, Xin Y. Mei, Ujash Sheth, Moin Khan, Darren de SA, Vehniah K. Tjong, Jesleen Rana

**Affiliations:** ^1^University of Toronto, Department of Surgery, 200 Elizabeth St, Toronto, ON M5G 2C, Canada; ^2^Queen's University, Kingston, ON, Canada; ^3^McMaster University, Department of Surgery, 280 Main Street West, Hamilton, ON L8S 4K1, Canada; ^4^Northwestern University, Department of Surgery, 676 N. St. Clair St., Suite 2320, Chicago, IL 60611, USA

## Abstract

**Background:**

The past two decades have seen a significant increase in consequences associated with nonmedical misuse of prescription opioids, such as addiction and unintentional overdose deaths. This study aimed to use an electronic survey to assess attitudes and opioid-prescribing practices of Canadian orthopaedic surgeons and trainees following open reduction internal fixation (ORIF) of distal radius and ankle fractures. This study was the first to assess these factors following ORIF of distal radius and ankle fractures using a survey design.

**Methods:**

A 40-item survey was developed focusing on four themes: respondent demographics, opioid-prescribing practice, patients with substance use disorders, and drug diversion. The survey was distributed among members of the Canadian Orthopaedic Association. Descriptive statistics were used to summarize respondent demographics and outcomes of interest. A Chi-square test was used to determine if proportion of opioid prescriptions between attending surgeons and surgeons in training was equal.

**Results:**

191 surveys were completed. Most respondents prescribed 10–40 tabs of immediate-release opioids, though this number varied considerably. While most respondents believed patients consumed only 40–80% of the prescribed opioids (73.6%), only 28.7% of respondents counselled patients on safe storage/disposal of leftover opioids. 30.5% of respondents felt confident in their knowledge of opioid use and mechanisms of addiction. Most respondents desired further education on topics such as procedure-based opioid-prescribing protocols (74.2%), alternative pain management strategies (69.7%), and mechanisms of opioid addiction (49.0%).

**Conclusions:**

The principle finding of this study is the lack of a standardized approach to postoperative prescribing in distal radius and ankle fractures, illustrated by the wide range in number of opioids prescribed by Canadian orthopaedic surgeons. Our data suggest a trend towards overprescription among respondents following distal radius and ankle ORIF. Future studies should aim to rationalize interventions targeted at reducing postoperative opioid prescribing for common orthopaedic trauma procedures.

## 1. Introduction

The opioid epidemic is a pressing public health issue in North America, affecting both orthopaedic surgeons and the general medical community alike [1]. While opioid medications have proven to be an effective method of postoperative analgesia, the past two decades have seen a significant increase in the negative societal consequences associated with nonmedical misuse of prescription opioids, such as harmful dependence and unintentional overdose deaths [2]. In Canada, the number of opioid-related deaths per 100,000 individuals has increased by 40% from 2016 to 2018 [3]. Similarly, the rate of hospitalization due to opioid overdose in Canada has increased by 53% from 2007 to 2017 [4]. In 2016 alone, Canada reported 8 opioid-related deaths and 16 opioid-related hospitalizations per day, numbers surpassing that of deaths secondary to motor vehicle collisions [5]. More recently, a report identified that opioid-related deaths increased by 38.2% in Ontario during the onset of the COVID-19 pandemic, illustrating the ever changing face of this epidemic [6].

Postoperative analgesia following orthopaedic surgery accounts for a substantial proportion of opioid prescriptions in Canada and the United States. In the United States, orthopaedic surgeons are the third-highest group of opioid-prescribing physicians, accounting for almost 8% of all opioid prescriptions in the country [7]. Similarly, Canadian orthopaedic surgeons have been identified as the second-highest group of opioid prescribers among Canadian physicians [8]. Moreover, the initial opioid prescription following orthopaedic procedures has been shown to account for an estimated 8.8% of all opioid-naïve patients undergoing surgery who subsequently develop chronic opioid dependence [9]. Furthermore, data from a 2019 study revealed that an average of 261 ± 154 (mean ± SD) milligram morphine equivalents (MME) were dispensed after a noninvasive meniscectomy in Canadian patients [10]. This is in the context of recent literature demonstrating satisfactory pain/patient outcomes postmeniscectomy with no postoperative opioid analgesia [11]. As a result of the apparent paradigm of overprescription, both the American Academy of Orthopaedic Surgeons (AAOS) and the Canadian Orthopaedic Association (COA) have published position statements to address the critical role that the orthopaedic surgeons must play in combating the opioid epidemic [12, 13].

Previous studies on postoperative opioid prescriptions following common orthopaedic procedures, such as lower extremity arthroplasty and arthroscopy, have demonstrated substantial variations in opioid-prescribing practices among orthopaedic surgeons, as well as a trend towards overprescription [14, 15]. The majority of these studies characterize prescribing patterns following elective orthopaedic procedures [14, 15]. Unfortunately, there remains a scarcity of literature on opioid-prescribing practices following trauma procedures. Moreover, the JBJS Pain Management Research Symposium identified several unique challenges in orthopaedic trauma, including the limited ability to modify preoperative risk factors such as opioid or drug use, and the unresolved concerns about the effects of NSAIDs on fracture healing [16]. Thus, many authors have called for additional studies investigating opioid-prescribing practices following trauma orthopaedic procedures to better understand current practices and to help establish standardized opioid-prescribing protocols.

This study therefore aims to use an anonymous electronic survey to assess the attitudes and opioid-prescribing practices of Canadian orthopaedic surgeons and trainees following open reduction internal fixation (ORIF) of distal radius and ankle fractures, two commonly performed orthopaedic trauma procedures. We hypothesized that, similar to the available Canadian and American data concerning elective orthopaedic procedures [14, 15], opioid-prescribing practices following distal radius and ankle ORIF would display significant variations among surveyed surgeons with a trend towards overprescription.

## 2. Materials and Methods

### 2.1. Survey Design

We assembled a focus group consisting of an author with experience in biostatistics, an addictions medicine specialist, and five fellowship-trained Canadian orthopaedic surgeons who routinely performed distal radius and ankle ORIF. The criteria for focus group selection was past publication and/or current vested interest literature concerning the opioid epidemic and significant experience with common orthopaedic trauma procedures at both the trainee (resident or fellow) and attending level. The web-based survey was drafted and finalized with input from all members of the focus group to optimize content clarity and comprehensiveness. The final 40-item survey included multiple choice questions, Likert scales, ranking questions, and open response questions focusing on four main themes: (1) respondent demographics, (2) opioid-prescribing practice, (3) patients with substance use disorders, and (4) drug diversion. A copy of the survey is available for review (see supplementary file (available here)). This study received institutional Research Ethics Board (REB) approval from Michael Garron Hospital on March 31, 2021.

### 2.2. Survey Administration

The survey was electronically distributed among all physician members of the Canadian Orthopaedic Association (COA) via email using the Survey Monkey platform (Palo Alto, CA) on May 6, 2021. One- and three-month reminder emails were sent on June 9 and August 5, 2021, respectively. Responses to the survey were closed on September 2021.

Survey responses were restricted to one per individual. Informed consent was obtained on the initial page of the survey. The survey had logic built-in such that if informed consent was not obtained, the respondent was directed to the closing remarks of the survey and was not permitted to complete the questions. Surgeons in training such as residents and clinical fellows were included in the study as they account for a large proportion of opioid prescriptions [17]. Following the end date of the survey, the raw data were exported to a secure Excel spreadsheet prior to statistical analysis.

### 2.3. Statistical Analysis

Descriptive statistics were used to summarize respondent demographic characteristics and outcome measures of interest. A Chi-square test was used to determine whether the proportion of quantity (ranges) of opioid prescriptions between attending surgeons and surgeons in training was equal. A Chi-square test was chosen due to the nature of the data output from the survey. Many questions were expressed in ranges rather than discreet values, which necessitated the use of a nonparametric alternative to a standard independent samples *T*-test. Bivariate correlation was used to examine potential relationships between factors (1) respondent gender, (2) years of practice, and (3) practice location on quantity of opioids prescribed. A *p* value <0.05 was considered statistically significant. All statistical analyses were performed using GraphPad Prism 8.0 (GraphPad Software, San Diego, CA).

## 3. Results

### 3.1. Respondent Demographics

The survey was successfully delivered to 621 of the total 1313 COA members. A total of 191 surveys were completed (30.7% response rate). Most respondents were male (79.5%) attending orthopaedic surgeons (63.8%) practicing in Ontario (58.7%), with an even distribution between academic and community practices. Surgical residents and clinical fellows comprised 29.7% and 5.4% of respondents, respectively. There was an even distribution in years of training among surgical residents. Detailed demographic information of the respondents is reported in Table 1.

### 3.2. Opioid-Prescribing Practice

Nearly all respondents prescribed immediate-release opioids following ankle and distal radius ORIF (98.2% and 97.6%, respectively), with the most common medication prescribed being one milligram hydromorphone tablets (48.5% and 45.1% for ankle and distal radius ORIF, respectively). Percocet (oxycodone/acetaminophen 5 mg/325 mg), Tylenol #3 (codeine/acetaminophen 30 mg/300 mg), and Tramacet (tramadol/acetaminophen 37.5 mg/325 mg) tablets were also commonly prescribed (18.7%, 17.5%, and 18.1% for ankle ORIF and 18.6%, 19.2%, and 21% for distal radius ORIF). Prescription of long-acting opioids is exceedingly rare (1.8% for either procedure). Commonly used perioperative analgesic adjuncts include Tylenol (74.1%), local anesthetic infiltration (71.8%), regional nerve block (39.1%), prescription NSAIDs (37.4%), and cryotherapy (19.5%).

**Figure 1:**
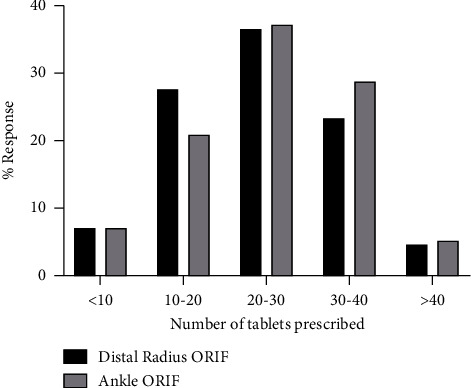
Opioid tablets prescribed following distal radius and ankle ORIF. % response: percentage of respondents.

The quantity of immediate-release opioids prescribed varied among the respondents for both procedures, though the majority prescribed 10–40 tabs. The proportion of respondents prescribing 10–20 tabs, 20–30 tabs, and 30–40 tabs of immediate-release opioid medication was 21.1%, 37.4%, and 28.9%, respectively, following ankle ORIF, and 27.7%, 36.8%, and 23.5%, respectively, following distal radius ORIF. This information is summarized in Figure 1. The Chi-square test did not demonstrate any differences in the proportion of quantity of opioids prescribed between attending surgeons and surgeons in training for both procedures *X*^2^ (12, 55) = 7.10, *p*=0.850. Moreover, the bivariate correlations performed did not reveal any significant relationships between gender, years of practice, and practice location on quantity of opioids prescribed (*p* > 0.05).

The respondents also varied in their estimation of the proportion of prescribed opioids that patients consume. Most respondents (73.6%) believe patients consume 40–80% of the prescribed opioids. Only 13.8% of respondents believe patients consumed 80–100% of the prescribed opioids. Only 28.7% of respondents counselled patients on safe storage and disposal of leftover opioids. Finally, there was no consensus regarding how long postoperatively orthopaedic surgeons should remain as the primary provider for pain management before referring back to their family physician or a pain specialist (2 weeks to >6 months).

### 3.3. Patients with Substance Use Disorder

Fifty-two percent of respondents routinely screened new patients for prior diagnosis of substance use disorder, and 89.9% altered their opioid-prescribing practices for patients with a positive history. The most common alterations in opioid-prescribing practice include prescribing less addictive opioid agents (53.3%), consulting another healthcare professional for recommendations (48.7%), and decreasing the number of refills (31.8%). Two-thirds (66.3%) of respondents believed that fewer than five percent of patients go on to develop chronic drug use problems after their orthopaedic care.

Interestingly, 61.1% of respondents reported encountering prescription fraud in their practice, with the most common methods being unsubstantiated claims of lost/stolen prescriptions (68.0%), heightened pain symptoms (55.3%), and forged prescriptions (35.9%). Common strategies used to combat prescription fraud include refusal to prescribe refills (59.6%), keeping copies of opioid prescriptions in the chart (47.5%), and faxing paper prescriptions directly to pharmacy (29.3%).

### 3.4. Factors Influencing Prescribing Practice

Fifty-seven percent of respondents have received supplemental training on opioid-prescribing. Despite this, only 30.5% of respondents feel confident or very confident in their knowledge of opioid use and mechanisms of addiction. Most respondents desired further education on opioid prescription and postoperative pain management, with the most requested topics being procedure-based opioid-prescribing protocols (74.2%), alternative pain management strategies (69.7%), and mechanisms of opioid overuse/addiction (49.0%). Factors deemed by respondents as most influential in determining their postoperative prescribing practices include personal experience with the procedure performed (36.5%) and attending surgeon preference (23.6%). Only 14.4% of respondents selected current evidence/literature as the most influential factor guiding their analgesic prescribing practices.

## 4. Discussion

Opioid-based analgesia following orthopaedic surgery has been identified as an important contributor to the ongoing opioid epidemic [7, 8]. As a result, increased efforts are made to better understand current opioid prescription practices to guide the creation of standardized opioid prescription protocols for common orthopaedic procedures. While recent studies have demonstrated substantial variations in opioid-prescribing practices following common elective orthopaedic procedures [14, 15], similar publications looking at common orthopaedic trauma procedures are lacking. Our study therefore aimed to assess the attitudes and opioid-prescribing practices of Canadian orthopaedic surgeons following distal radius and ankle ORIF, two of the most common orthopaedic trauma procedures.

The current study demonstrated variations in both the quantity and type of opioid medications prescribed following distal radius and ankle ORIF. While most respondents prescribed between 10 and 40 tabs of immediate-release opioids postoperatively, responses were split evenly between the 10–20 tabs, 20–30 tabs, and 30–40 tabs categories, suggesting a lack of consensus regarding the optimal quantity to prescribe. To better illustrate this distribution in context with two of the most prescribed opioids after ankle and distal radius ORIF (Percocet, oxycodone 5 mg and Dilaudid, hydromorphone 1 mg), this would represent a range of 75–300 and 40–160 morphine milligram equivalent (MME), respectively. The quantity of opioids prescribed in the first prescription is similar to the American literature-reported values for both distal radius and ankle ORIF and lower than that of elective orthopaedic procedures [18, 19]. Respondents in our survey also prescribed both “strong” opioids such as hydromorphone and oxycodone (contained in Percocet), as well as codeine (contained in Tylenol #3) and tramadol (contained in Tramacet), sometimes referred to as “weak” opioids. This is consistent with findings from previous studies looking at elective orthopaedic procedures [20]. Additionally, three-quarters of respondents estimated that only 40–80% of patients fully consumed their prescribed opioids. Together, these findings suggest that similar to our American colleagues, Canadian orthopaedic surgeons also display a lack of consensus and self-identified trend towards opioid overprescription. Furthermore, the variation in the quantity and type of opioids prescribed highlights the need for standardized opioid-prescribing guidelines for common orthopaedic procedures. Such guidelines would complement a patient-centered, individualized approach to treating postoperative pain. The implication of the observed, considerable variations and lack of consensus in opioid-prescribing practices is the potential for over prescription and drug diversion as outlined in an editorial by Makary et al. [21].

Our study also identified several areas for improvement to minimize opioid diversion and increase physician confidence in safe opioid-prescribing practices. First, routine patient counselling on safe disposal of unused opioid medications may help decrease opioid diversion and subsequent nonmedical misuse. While three-quarters of surgeons in our study estimated 40–80% consumption of the prescribed opioids, only one quarter of surgeons routinely counselled patients on how to safely dispose unused opioid medications. This is particularly concerning given that 61.1% of respondents experienced prescription fraud in their practice. The discrepancy between physician prescription and patient consumption of opioids following orthopaedic surgery is well-reported in the literature. A 2018 study analyzing 1,199 orthopaedic procedures showed that patients were prescribed an excess of pills approximately 60% of the time [22]. Another 2016 study involving 1,416 upper extremity procedures found that patients on average consumed only 34% of the prescribed opioids [23]. Given that counselling has been strongly associated with increased safe disposal of unused opioids by patients [24], orthopaedic surgeons may often have the first opportunity to mitigate the harm from diversion of unused opioid medications by routinely counselling patients on safe disposal strategies [25]. Second, optimizing perioperative multimodal analgesia may help decrease postoperative opioid requirements. While there is ongoing controversy regarding whether the use of NSAIDs increases the risk of nonunion and delayed union, Tylenol, regional blocks, and local anesthetic infiltration are relatively low-risk perioperative analgesic adjuncts that have been shown to decrease postoperative opioid consumption [26–28]. Thus, standardized perioperative multimodal analgesia protocols should be established to include these adjuncts when no clear contraindications exist. Lastly, supplemental training on safe opioid-prescribing practices may increase the confidence of the prescribing surgeons, particularly given that many respondents have identified prescription fraud as source of concern. In our study, less than one-third of respondents felt confident in their knowledge of opioid use and mechanisms of addiction, though most were open to supplemental education and other opioid safety initiatives. Similar educational initiatives have been shown to increase the comfort of primary care physicians in safe opioid-prescribing [29]. Furthermore, preoperative patient education has also been shown to decrease postoperative opioid consumption [30]. Finally, evidence-based, procedure-specific opioid prescription guidelines in general surgery and gynecological literature have been shown to decrease opioid prescription dramatically while providing adequate analgesia [31]. It is likely that educational initiatives in these areas will show similar benefit in the orthopaedic setting.

This study has several limitations. First, our survey response rate (30.7%) was moderate, thus introducing the potential for selection bias. Possible reasons for lack of response include lack of desire to participate in online surveys and lack of involvement in distal radius and ankle ORIF. However, the considerable distribution between 10–20, 20–30, and 30–40 tablets postdistal radius and ankle ORIF would suggest that we did not select a biased sample. Moreover, our survey did attain a robust sample size with an even distribution between attending surgeons and surgeons in training, which increases the generalizability of our results. Second, this is a self-report survey study and not an observational study. The results are therefore based on surgeons' opinions regarding postoperative analgesia, while patient-reported pain scores and attitudes were not collected. Thus, there is a potential for self-report bias in this study. Furthermore, the aim of this study was to elucidate both the attitudes and practices of Canadian orthopaedic surgeons; thus, the objectivity of this study is limited on purpose and by design. In the design phase, we aimed to create a survey that would not elucidate leading questions and subsequent responses. Nonetheless, there remains the possibility that response bias was present in this study. Lastly, our study did not explore how the identified opioid-prescribing patterns relate to actual narcotic prescription filling or consumption.

## 5. Conclusion

Our investigation demonstrated wide variations in opioid-prescribing practices, as well as a trend towards overprescription among Canadian orthopaedic surgeons following distal radius and ankle ORIF. Our survey also identified several target areas for future initiatives focused on improving opioid-prescribing practices. These include routine patient counselling on safe disposal of unused opioid medications, optimizing perioperative multimodal analgesia, and supplemental training on safe opioid-prescribing practices, including the development of standardized opioid-prescribing guidelines for common orthopaedic procedures. Future studies should aim to rationalize interventions targeted at reducing postoperative opioid prescribing for common orthopaedic trauma procedures.

## Figures and Tables

**Table 1:** Respondent demographics.

	*n*	% of respondents
*Age (yrs)*		
<30	31	16.7
30–39	68	36.8
40–49	44	23.8
50–59	25	13.5
>60	17	9.2

*Sex*		
Male	147	79.5
Female	36	19.5

*Position*		
Attending	118	63.8
Clinical fellow	10	5.4
Surgical resident	55	29.7

*Type of practice (attendings)*		
Academic	51	38.9
Community	58	44.3
Mixed	21	16.0

*Years in practice (attendings)*		
<5	32	25.0
5–10	29	22.7
10–15	23	18.0
15–20	10	7.8
>20	34	26.6

*Level of training (residents)*		
PGY-1	21	38.2
PGY-2	7	12.7
PGY-3	8	14.6
PGY-4	6	10.9
PGY-5	13	23.6

*n*: number of respondents; PGY: postgraduate year.

## Data Availability

The data used in this study are available upon request from the author.

## Supplementary Materials

Supplementary Materials Copy of Survey Disseminated to Respondents.
