# Supplementary material for: Attitudes and Practices Surrounding Opioid Prescriptions following Open Reduction Internal Fixation of Distal Radius and Ankle Fractures: A Survey of the Canadian Orthopaedic Association Membership
Source: Adv Orthop. 2023 Sep 7;2023:9968219. doi: 10.1155/2023/9968219 (PMC10501842; doi:10.1155/2023/9968219)
Supplement: Supplementary Materials — Copy of Survey Disseminated to Respondents. [file 9968219.f1.pdf]

MICHAEL GARRON HOSPITAL
TORONTO EAST HEALTH NETWORK

OPIOID PRESCRIBING ATTITUDES AND PRACTICES OF CANADIAN ORTHOPEDIC SURGEONS IN POST-OPERATIVE PAIN MANAGEMENT OF DISTAL RADIUS AND ANKLE FRACTURES

Full Questionnaire

MICHAEL GARRON HOSPITAL
TORONTO EAST HEALTH NETWORK

SECTION A: DEMOGRAPHICS

Check the box that BEST represents you and/or your practice

1. A) Male B) Female C) No response

2. Your age (please list)______________

3. Please select which best describes your position
   A) Staff Surgeon/Attending
   B) Clinical Fellow
   C) Locum Physician
   D) Resident

4. **(Staff Surgeons/Attendings and Locum Physician)** How many years have you been practicing? _____________

5. (**Residents)** What year of training are you in (e.g. PGY-2)? _________________

6. Which Province do you currently practice or train in? (please list) ____________

7. **(Staff Surgeons/Attendings)** Where is your practice located?
   A) Academic centre
   B) Community hospital
   C) Both of above
   D) Other

SECTION B: PAIN PRESCRIBING PRACTICE

1. In your current practice or training, do you perform **ankle fracture surgery (i.e. ORIF)**? A) Yes B) No

2. If yes, do you prescribe immediate release opioids? A) Yes B) No

3. If so, which opioids do you most often prescribe for this procedure?
   A) Hydromorphone

MICHAEL GARRON HOSPITAL
TORONTO EAST HEALTH NETWORK

B) Oxycodone
C) Codeine
D) Tramadol
E) Morphine Sulfate
F) Other (please list) ______________

4. How many pills and at what doses do you prescribe for this procedure? Please list (range is acceptable, example: Percocet, 20-30 pills at 5mg)

5. Do you prescribe long acting opioids for this procedure? A) Yes B) No

6. If yes, which opioids do you most often prescribe for this procedure?

   A) Morphine
   B) Fentanyl
   C) Oxycodone (controlled release)
   D) Methadone
   E) Other (please list) ________________

7. How many pills and at what doses do you prescribe for this procedure? Please list (range is acceptable)

8. In your current practice or training, do you perform **distal radius fracture surgery (i.e. ORIF)**?
   A) Yes B) No

9. If yes, do you prescribe immediate release opioids? A) Yes B) No

10. If so, which opioids do you most often prescribe for this procedure?
   A) Hydromorphone
   B) Oxycodone
   C) Codeine
   D) Tramadol
   E) Morphine Sulfate
   F) Other (please list) ______________

11. How many pills and at what doses do you prescribe for this procedure? Please list (range is acceptable, example: Percocet, 20-30 pills at 5mg)

12. Do you prescribe long acting opioids for this procedure? A) Yes B) No

13. If yes, which opioids do you most often prescribe for this procedure?

   A) Morphine

B) Fentanyl
C) Oxycodone (controlled release)
D) Methadone
E) Other (please list) ________________

14. How many pills and at what doses do you prescribe for this procedure? Please list (range is acceptable)

15. What adjuncts do you routinely use perioperatively with opioids? Check ALL that apply. (**residents/fellows may select options they routinely see used**)
A) Nerve block
B) Prescription NSAID
C) Local anesthetic
D) Intra-articular injection (e.g. Cortisone or Morphine)
E) Cryotherapy
F) Over the counter pain meds (Ibuprofen, ASA, Acetaminophen, etc)
G) Neuromodulators (Gabapentin, Pregabalin)
H) Other (please list) _____________
I) Not Applicable

16. Estimate the percentage of your patients that you granted a refill or alternate opioid prescription (either in fracture clinic or a call to surgeon's office)
A) 0-20%
B) 20-40%
C) 40-60%
D) 60-80%
E) 80-100%

17. Do you counsel your patients on safe storage and disposal of leftover opioids?
A) Yes B) No

18. On average, how much of their total dispensed pain medications do you believe your patients are taking?

A) (0% to 20%)
B) (20% to 40%)
C) (40% to 60%)
D) (60% to 80%)
E) (80% to 100%)

MICHAEL GARRON HOSPITAL
TORONTO EAST HEALTH NETWORK

19. (**Staff Surgeons/Attendings)** How long do you continue to manage your patients' pain before referring them back to a pain specialist or primary physician?
    A) 2 weeks
    B) 4 weeks
    C) 6 weeks
    D) 12 weeks
    E) 6 months or longer

SECTION C: PATIENTS WITH DRUG ADDICTION/ABUSE ISSUES

1. When treating a new patient, do you ask the patient if he/she has a history of drug addiction or substance abuse? A) Yes B) No

2. Do you alter your prescribing practice of opioid analgesics if a patient acknowledges he/she has a drug addiction? A) Yes B) No

3. If yes, which of the following statements best describes how you change your prescription of opioid analgesics? Check ALL that apply.

    A) Refusal to prescribe opioid
    B) Prescribe less-addicting agent
    C) Decrease the dosage of opioid
    D) Recommend that a partner control their medication
    E) Increase the dosage due to potential tolerance
    F) Decrease the number of refills
    G) Consult another healthcare professional for recommendation (e.g. pharmacist)

4. Estimate the percentage of patients who go onto developing chronic drug use/addiction problems after your treatment (range is acceptable) ___________

5. Do you believe you have been the victim of prescription fraud or theft?

    A) Yes B) No

6. If yes, how have you been victimized? (Check ALL that apply)

MICHAEL GARRON HOSPITAL
TORONTO EAST HEALTH NETWORK

A) Fake pain symptoms
B) Forged written prescriptions
C) Fake prescription phone-ins
D) Altered number of refills
E) Stolen prescription pads
F) Stolen medication from office
G) Altered pill quantity
H) Patient claims lost or stolen prescription
I) Fabricated symptoms (e.g purposefully dislocating shoulder to get prescription)

7. If you have been victimized, how have you changed your prescription protocol (please describe) _______________

SECTION D: DRUG DIVERSION - FOR THE FOLLOWING QUESTIONS CONSIDER YOUR PAIN MANAGEMENT EXPERIENCE.

1. At any point in your training or practice, have you received any supplemental education or training on opioid prescribing?
 A) Yes B) No

2. Do you feel confident in your knowledge of opioid use and mechanisms of overuse/addiction when consulting with your patients? Use the following scale to respond.

Not confident at all		Somewhat confident		Very confident
1	2	3	4	5

3. If you responded from 1 (Not confident) to 3 (somewhat confident), which area do you feel education or proficiency would aid in your confidence?
A) Mechanisms of overuse/addiction
B) Alternative pain management techniques
C) Procedure-based optimal prescriptions
D) Other (please list) ___________

MICHAEL GARRON HOSPITAL
TORONTO EAST HEALTH NETWORK

4. Which of the following factors are most influential in your post-operative analgesic prescribing behaviours? Please rank options in order of importance to your particular preference, with (1) being most important (2) being second most important, and so forth.

   You may rank all options, but please rank at least 1. If a choice does not play a role in your decision, select NA (not applicable)

   A) Personal experience with the procedure performed
   B) Maximize patient satisfaction and minimize dissatisfaction
   C) Current evidence or literature relative to procedure performed
   D) Attending surgeon preference
   E) The same medication, dosage, and number of pills prescribed for all patients undergoing a given surgical procedure
   F) Concern that patient will run out of medication or to avoid having to re-prescribe prior to the next visit

5. Are you aware of any local acute or chronic pain monitoring and management programs?
   A) Yes
   B) No
6. If yes, estimate the amount of times in the past year have you referred your patients to this kind of program? (please list) ______________

SECTION E: QUESTIONS AND COMMENTS

1. Any questions/comments for the authors?
   ____
